# SYMBALS: A Systematic Review Methodology Blending Active Learning and Snowballing

**DOI:** 10.3389/frma.2021.685591

**Published:** 2021-05-28

**Authors:** Max van Haastrecht, Injy Sarhan, Bilge Yigit Ozkan, Matthieu Brinkhuis, Marco Spruit

**Affiliations:** ^1^ Department of Information and Computing Sciences, Utrecht University, Utrecht, Netherlands; ^2^ Department of Computer Engineering, Arab Academy for Science, Technology and Maritime Transport (AASTMT), Alexandria, Egypt; ^3^ Department of Public Health and Primary Care, Leiden University Medical Center (LUMC), Leiden, Netherlands; ^4^ Leiden Institute of Advanced Computer Science (LIACS), Leiden University, Leiden, Netherlands

**Keywords:** systematic review, methodology, active learning, machine learning, backward snowballing

## Abstract

Research output has grown significantly in recent years, often making it difficult to see the forest for the trees. Systematic reviews are the natural scientific tool to provide clarity in these situations. However, they are protracted processes that require expertise to execute. These are problematic characteristics in a constantly changing environment. To solve these challenges, we introduce an innovative systematic review methodology: SYMBALS. SYMBALS blends the traditional method of backward snowballing with the machine learning method of active learning. We applied our methodology in a case study, demonstrating its ability to swiftly yield broad research coverage. We proved the validity of our method using a replication study, where SYMBALS was shown to accelerate title and abstract screening by a factor of 6. Additionally, four benchmarking experiments demonstrated the ability of our methodology to outperform the state-of-the-art systematic review methodology FAST^2^.

## 1 Introduction

Both the number of publishing scientists and the number of publications are constantly growing (Ware and Mabe, 2015). The natural scientific tool to provide clarity in these situations is the systematic review (Glass, 1976), which has spread from its origins in medicine to become prevalent in a wide number of research areas (Petticrew, 2001). Systematic reviews offer a structured and clear path to work from a body of research to an understanding of its findings and implications (Gough et al., 2017; Higgins et al., 2019). Systematic reviews are ubiquitous in today’s research. A search in the Scopus abstract database for the phrase “systematic review” yields more than 45,000 results for the year 2020 alone.

Nevertheless, systematic reviews have shortcomings. They are particularly protracted processes (Borah et al., 2017; O’Connor et al., 2019), that often require an impractical level of expertise to execute (Zhang and Ali Babar, 2013). These issues have been recognised for decades (Petticrew, 2001), but not solved. This hampers our ability as researchers to apply this potent tool in times where change is ceaseless and sweeping.

However, with recent advances in machine learning and active learning, new avenues for systematic review methodologies have appeared (Marshall and Wallace, 2019). This is not to say that these techniques make traditional systematic review techniques obsolete. Methodologies employing automation techniques based on machine learning are often found to omit around 5% of relevant papers (Yu et al., 2018b; Gates et al., 2019; Yu and Menzies, 2019). Additionally, usability and accessibility of automation tools is a common issue (Gates et al., 2019; Harrison et al., 2020) and many researchers do not trust machine learning methods enough to fully rely on them for systematic reviews (O’Connor et al., 2019).

Therefore, in this paper, we argue for the combination of the proven method of backward snowballing (Wohlin, 2014) with novel additions based on machine learning techniques (van de Schoot et al., 2021). This yields SYMBALS: a SYstematic review Methodology Blending Active Learning and Snowballing. The challenges faced by systematic review methodologies motivate the research question of our paper:

How can active learning and snowballing be combined to create an accessible and swift systematic review methodology?

The remainder of this paper is structured as follows. In Section 2, we cover related work on systematic review methodologies and active learning techniques for systematic reviews. In Section 3, we introduce SYMBALS, our innovative systematic review methodology. We explain each step of the methodology in detail. Section 4 evaluates and demonstrates the effectiveness of our methodology using two case studies: a full application of SYMBALS 4.1 and a benchmarking study 4.2. In Section 5, we discuss the implications of the case studies and the limitations of our research. Finally, we conclude and present ideas for future research in Section 6.

## 2 Related Work

### 2.1 Systematic Review Methodologies

From its origins (Glass, 1976) and main application in the field of medicine, the use of systematic reviews has spread across the research community (Petticrew, 2001). In the area of information systems, the use of this tool was limited only 2 decades ago (Webster and Watson, 2002). Yet, systematic reviews are ubiquitous in the field now.

Software engineering is a field of research that has been specifically active in propelling systematic review practice. Since the first push for Evidence-Based Software Engineering (EBSE (Kitchenham et al., 2004)), many contributions to systematic review practice have been made. Learning from applying the process in their domain (Brereton et al., 2007), clear guidelines for performing systematic reviews were developed (Kitchenham and Charters, 2007). These guidelines have been implemented and new methodologies have been developed and formalised. An example is the snowballing methodology (Wohlin, 2014).

Hybrid strategies have emerged which combine results from abstract databases with snowballing (Mourão et al., 2017, 2020), as well as those that suggest automating certain steps of the systematic review process with machine learning techniques (Osborne et al., 2019). The use of systematic reviews in software engineering has matured to a stage where even tertiary studies—reviews of reviews—are common (Kitchenham et al., 2010). These studies focus on issues such as orientation towards practice (da Silva et al., 2011), quality evaluation (Khan et al., 2019), and time investment (Zhang and Ali Babar, 2013). Tertiary studies give insight into what constitutes a high-quality systematic review. We used these insights in constructing our methodology.

Even with all of the developments in systematic review methodologies, challenges remain. At the heart of these challenges lie the tradeoffs between automation and completeness and between automation and usability. Approaches using automation techniques to speed up the systematic review process generally miss approximately 5% of the relevant papers that would have otherwise been found (Yu et al., 2018b; Gates et al., 2019; Yu and Menzies, 2019). Additionally, many automation tools for systematic reviews still suffer from usability issues. Some tools are evaluated as hard to use (Gates et al., 2019), while others are not suitable due to limited accessibility (Harrison et al., 2020).

The usability issues are certainly solvable. Certain automation tools already offer a good user experience (Harrison et al., 2020) and some are making their code available open-source (van de Schoot et al., 2021), making these tools increasingly accessible and transparent. The concerns regarding completeness remain. However, we should be aware that the metric used to assess completeness - the percentage of the total relevant papers found using an automated process (Gates et al., 2019)—is quite strict. The metric assumes that the complete set of relevant papers were found in the original review, meaning the automated method can at best perform equally well.

With SYMBALS we advocate for the adoption of usable and accessible automation tools, specifically those facilitating active learning for title and abstract screening. By combining automation with backward snowballing, we hope to address the completeness concerns that are still prevalent in many fully automated methods. Given the relative novelty and complexity of active learning techniques, we opt to provide further explanation and contextualisation of active learning in Section 2.2.

### 2.2 Active Learning for Systematic Reviews

Active learning is a machine learning method whereby a learning algorithm chooses the most relevant data points to learn from. The key concept motivating this approach is that the algorithm will perform better with fewer training samples if it can guide the learning process towards the most informative samples (Settles, 2012). This makes it very well suited to be applied in the title and abstract screening phase of systematic reviews, where researchers often start with a large set of papers and prefer to not perform the full time-consuming task manually (Yu et al., 2018b).

Active learning for title and abstract screening works as follows. Researchers construct a dataset of potentially relevant research, with at least a title and abstract for each paper. Researchers should then define an initiation process and an appropriate stopping criterion for the active learning algorithm. The exact initiation process will differ, but the initial sample provided to the algorithm should contain at least one relevant and one irrelevant paper for the algorithm to learn from. At the same time, the sample should be relatively small compared to the complete set of papers, as there is no time advantage in this phase of the process.

**FIGURE 1:**
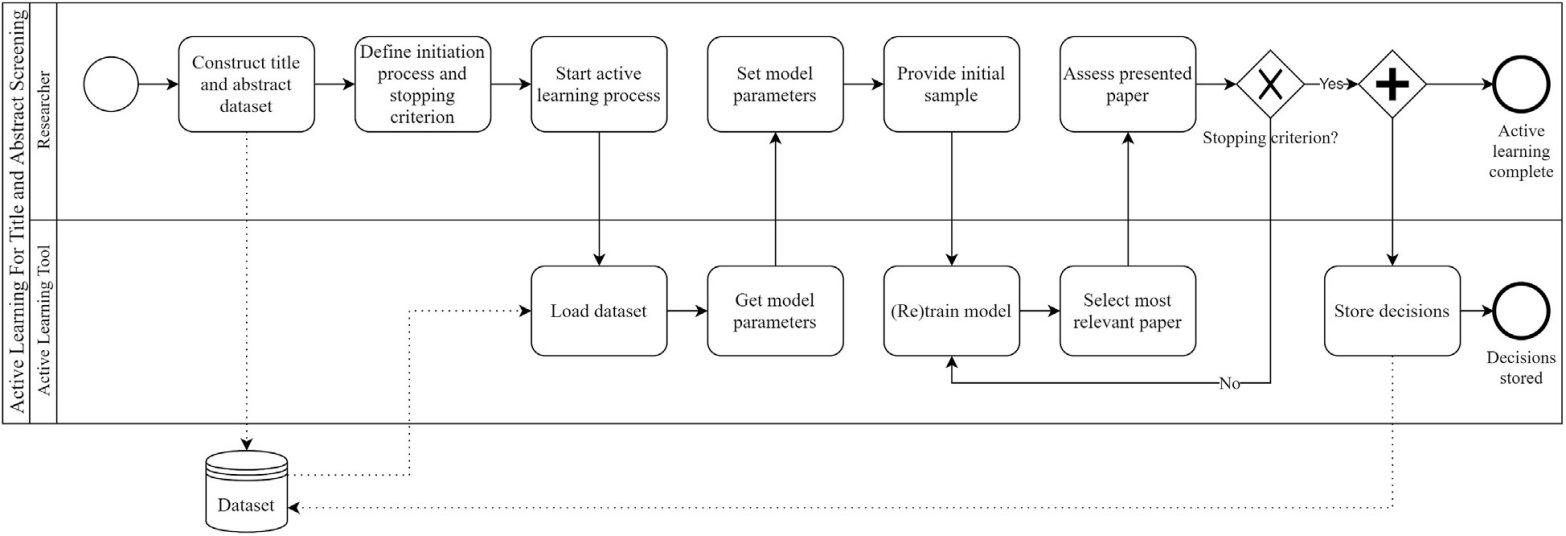
The active learning for title and abstract screening process, depicted using BPMN. One can clearly see why this process is characterised as “researcher-in-the-loop” (van de Schoot et al., 2021) machine learning.

After the algorithm has learned from the initial samples, it will present the researchers with the most informative paper first (Yu and Menzies, 2019). The researcher indicates whether the paper is relevant or irrelevant and the algorithm uses this input to retrain. The key challenge is to balance exploration and exploitation. The algorithm should learn to distinguish relevant from irrelevant papers as quickly as possible (exploration) while presenting the researchers with as many relevant papers as possible (exploitation). Active learning techniques have been shown to significantly reduce the time spent on title and abstract screening (Miwa et al., 2014), while minimally affecting the total number of relevant papers found (Yu et al., 2018b). Using active learning for title and abstract screening can intuitively be characterised as “researcher-in-the-loop” (van de Schoot et al., 2021) machine learning. Figure 1 depicts the active learning process using Business Process Model and Notation (BPMN).

In an evaluation of 15 software tools that support the screening of titles and abstracts (Harrison et al., 2020), Abstrackr (Wallace et al., 2012), Covidence (Babineau, 2014), and Rayyan (Ouzzani et al., 2016) emerged as the tools that scored best. FASTREAD (Yu et al., 2018b) and ASReview (van de Schoot et al., 2021) are two additional tools incorporating active learning that have recently been introduced.

The first research using active learning techniques to supplement systematic reviews is beginning to appear. For the steps of “identify research” and “select studies” (Kitchenham et al., 2015), some suggest using active learning on database results as the sole method (Yu and Menzies, 2019). This yields a fast approach, as seen with the FASTREAD (Yu et al., 2018b) and FAST^2^ (Yu and Menzies, 2019) methodologies. However, these methods sacrifice a degree of completeness to manual screening (Gates et al., 2019), which itself can omit up to 30% of the relevant papers that could have been found by additionally using other techniques than database search (Mourão et al., 2017, 2020).

Approaches relying solely on database search also have no way of incorporating grey literature. Grey literature is research that does not originate from traditional academic publishing sources, such as technical reports and dissertations. This issue could be solved by searching for grey literature before screening (Rios et al., 2020), although this requires the researchers to know where to find relevant grey sources. The issues relating to the completeness of the review can be solved by incorporating a backward snowballing phase after database searching and screening (Mourão et al., 2017, 2020), which is exactly what we suggest to do in our approach.

Active learning is not the only machine learning approach used to automate systematic reviews. Some researchers have suggested using natural language processing techniques to aid database search (Osborne et al., 2019; Marcos-Pablos and García-Peñalvo, 2020), while others prefer to use reinforcement learning in title and abstract screening, rather than active learning (Ros et al., 2017). However, with the prevalence of active learning systematic review tools (Harrison et al., 2020), active learning is at this point the most approachable machine learning method for systematic reviews, with the clearest benefits coming in the title and abstract screening phase (van de Schoot et al., 2021). By incorporating active learning, SYMBALS expedites the systematic review process while remaining accessible.

Table 1 compare provides an overview of the discussed papers that present a systematic review methodology. Methodologies that include automation techniques will generally be swifter, but accessibility can suffer. These methodologies can be less accessible due to their reliance on techniques and tooling that is not freely and publicly available, as is the case for the reinforcement learning approach of Ros et al. (2017). Additionally, since many researchers still do not fully trust automation techniques for systematic reviews (O’Connor et al., 2019), methodologies using these techniques are less accessible in the sense of being less approachable. One way to solve this issue is to incorporate trusted systematic reviews methods such as snowballing, as we propose to do with SYMBALS. Table 1 shows that a methodology that manages to be both accessible and swift is unique. Therefore, if SYMBALS manages to foster accessibility and swiftness, it has the potential to be of added value to the research community.

**TABLE 1:** Overview of systematic review methodologies discussed in Section 2, the methods they use, and the properties they possess.

	Methods			Properties	
Research	DB search	Automation	Snowballing	Accessible	Swift
SYMBALS	✓	✓	✓	✓	✓
Miwa et al. (2014)	✓	✓	×	×	✓
Wohlin (2014)	×	×	✓	✓	×
Ros et al. (2017)	✓	✓	✓	×	✓
Mourão et al. (2017)	✓	×	✓	✓	×
Yu et al. (2018a)	✓	✓	×	×	✓
Yu and Menzies (2019)	✓	✓	×	×	✓
Mourão et al. (2020)	✓	×	✓	×	✓
Rios et al. (2020)	✓	✓	×	×	✓

## 3 SYMBALS

**FIGURE 2:**
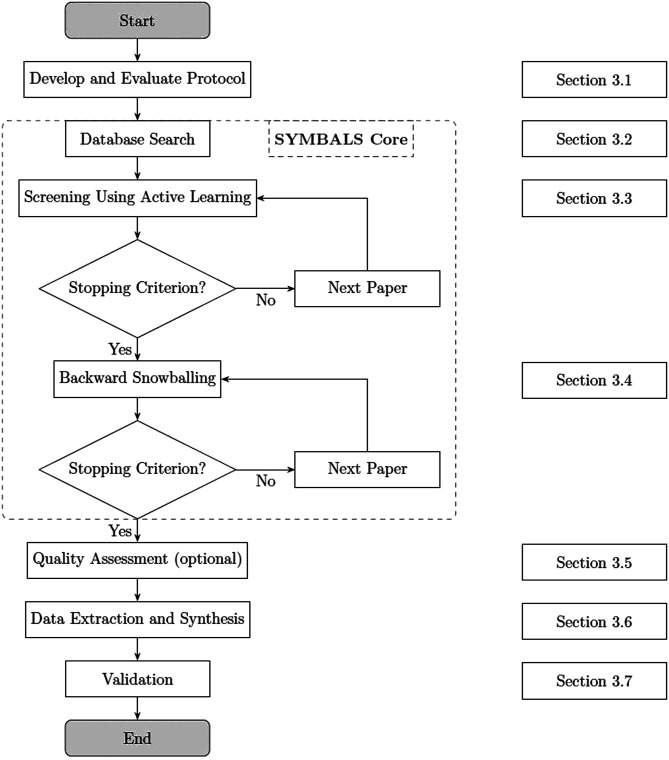
SYMBALS, our proposed systematic review methodology. The methodology consists of the SYMBALS core (dashed box), supplemented with elements of the stages of planning and conducting a review (Kitchenham and Charters, 2007).

In this paper, we introduce SYMBALS: a SYstematic review Methodology Blending Active Learning and Snowballing. Figure 2 presents our methodology. Focusing on the planning and conducting phases of a systematic review (Kitchenham and Charters, 2007), SYMBALS complements existing review elements with active learning and snowballing steps. The following sections outline the steps that together constitute SYMBALS.

### 3.1 Develop and Evaluate Protocol

Any systematic review is instigated from a motivation and a need for the review (Wohlin et al., 2012). These lead to the formulation of research questions and the design of a systematic review protocol (Kitchenham and Charters, 2007). A protocol for SYMBALS should contain the following items:

• Background, rationale, and objectives of the systematic review.
• Research questions the systematic review aims to answer.
• Search strategy to be used.
• Selection criteria to be applied.
• Selection procedure to be followed.
• Data extraction, management, and synthesis strategy.
• Validation method(s) used to validate the procedure and the results.

Quality assessment checklists and procedures (Kitchenham and Charters, 2007) are vital to include if one plans to apply a quality assessment step. However, it is recognised that this is not a necessary phase in all systematic reviews (Brereton et al., 2007). Additional items that can potentially be included in a protocol are the risks of bias in the primary studies and the review itself (Moher et al., 2015), as well as a project timetable and dissemination strategy (Kitchenham and Charters, 2007; Wohlin et al., 2012).

For researchers in the field of information systems and other comparable fields, it is important to be aware of two potential roadblocks to implementing our methodology. Firstly, not all databases are designed to support systematic reviews (Brereton et al., 2007), meaning researchers may need to apply different search criteria in different sources. Secondly, abstracts in the information systems field are often of a quality that is too poor to be relied upon when applying selection criteria (Brereton et al., 2007). This problem can be circumvented by additionally inspecting the conclusions of these papers, and we have not found this issue to extensively impact the effectiveness of the active learning phase of SYMBALS.

### 3.2 Database Search

Once researchers are content with their search string selection, they can start with the database search step of SYMBALS. Techniques exist to aid researchers in formulating their search query (Marcos-Pablos and García-Peñalvo, 2018), even involving machine learning methods (Marcos-Pablos and García-Peñalvo, 2020bib_Marcos_Pablos_and_García_Peñalvo_202). We highly recommend researchers consult these methods to help in swiftly constructing a suitable search string.

The advantage of SYMBALS is that the search string does not need to be perfect. Not all databases offer the same search capabilities (Singh and Singh, 2017), meaning that complex, tailor-made search queries are often not reproducible across databases (Mourão et al., 2017). By using active learning, the impact of including papers that should not have been included is minimised. Concurrently, backward snowballing limits the impact of excluding papers that should have been included. By facilitating the use of a broad search query, SYMBALS is accessible for researchers without extensive experience in the field being considered. This is not only a benefit to junior researchers and students but also to researchers looking to map findings from other areas to their field of interest.

Different databases are relevant in different disciplines, and the set of relevant databases is bound to change over time. This is the reason that we do not recommend a fixed set of databases for our approach. Nevertheless, a few points are worth noting regarding the choice of database. Generally, there is a consensus of which databases are relevant to a particular field (Kitchenham and Charters, 2007; Brereton et al., 2007), and research has shown which databases are suitable for systematic reviews (Gusenbauer and Haddaway, 2020). Additionally, researchers should be aware of the required data of the active learning tool they intend to use for screening.

### 3.3 Screening Using Active Learning

In the active learning phase, we recommend using existing and freely accessible active learning tools that are aimed at assisting title and abstract screening for systematic reviews. Researchers can consult tool evaluations (Harrison et al., 2020) to decide for themselves which tool they prefer to use. Although even the tools specifically aimed at automating systematic reviews suffer from a lack of trust by researchers (O’Connor et al., 2019), we believe that initiatives such as those to make code available open-source (van de Schoot et al., 2021) will solve many of the trust issues in the near future.

It is difficult to choose an appropriate active learning stopping criterion (Yu and Menzies, 2019). Some tools choose to stop automatically when the algorithm classifies none of the remaining papers as relevant (Wallace et al., 2012). Although this accommodates reproducibility, it is generally not acceptable for researchers to have no control over when they are done with their screening process. Commonly used stopping criteria are to stop after evaluating *n* irrelevant papers in a row or after having evaluated a fixed number of papers (Ros et al., 2017). The simplicity of these stopping criteria is pleasant, but these criteria are currently not considered best practice (Yu and Menzies, 2019).

Of particular interest are those criteria that are based on an estimate of the total number of relevant papers in the starting set (Cormack and Grossman, 2016). Let *N* be the total number of papers and *R* the number of relevant papers. In general, *R* is not known. To estimate *R* we can evaluate papers until we have marked *r* papers as relevant. Let *i* denote the number of papers that are marked as irrelevant at this stage. We can then estimate *R* as:

$$R\approx N\times \frac{r}{r+i}.$$ (1)

A potential stopping criterion is then to stop once a predefined percentage *p* of the estimated number of relevant papers *R* has been marked relevant. This criterion solves the issues that the earlier criteria faced. Implementations of this approach that are more mathematically grounded exist (Cormack and Grossman, 2016; Yu and Menzies, 2019), and we encourage researchers to investigate those methods to decide on their preference.

### 3.4 Backward Snowballing

There are systematic review methods that move straight to the quality assessment stage after applying active learning (Yu and Menzies, 2019). In SYMBALS we choose to blend active learning and backward snowballing. This allows researchers to complement their set of relevant papers with additional sources. There are three main classes of relevant papers that may not be included at this stage. The first is the group of relevant papers included in the set that was automatically excluded in the active learning phase. An appropriately defined stopping criterion should keep this set relatively small. Additionally, there are relevant papers that do not satisfy the search query used. Last, and certainly not least, is the group of relevant papers that are not present in the databases considered. This will mostly be grey literature and, from our experience, relatively old research.

Altogether these groups form the motivation to include a snowballing step, and it has been shown that this step has the potential to add many relevant papers, even after a database search (Mourão et al., 2020). Additional relevant research can be identified from the reference lists (backward snowballing) and citations (forward snowballing) of included papers (Wohlin, 2014). After constructing an initial set of relevant inclusions and defining a stopping criterion, the backward snowballing procedure begins. In SYMBALS, the set of inclusions to consider is the set originating from the active learning process. This set will generally be much larger than the initiating set of a regular snowballing procedure (Wohlin, 2014). This makes it vital to define a suitable stopping criterion, to prevent the backward snowballing process from taking up too much time.

**FIGURE 3:**
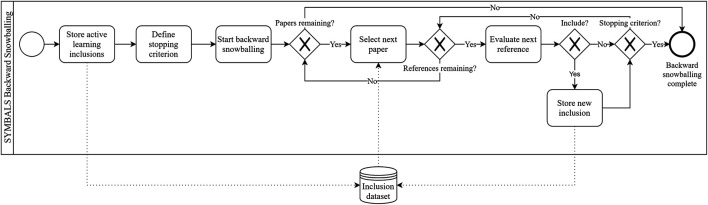
The backward snowballing process in the SYMBALS setting, depicted using BPMN. Although our process clearly differs from the traditional backward snowballing process, the diagram is undeniably similar to conventional snowballing diagrams (Wohlin, 2014).

Figure 3 depicts the backward snowballing procedure in our setting. The procedure differs from the traditional backward snowballing procedure (Wohlin, 2014) due to the large set of inclusions that already exist in our process from the active learning phase. This also implies the stopping criterion for backward snowballing has to differ from traditional stopping criteria (Wohlin, 2014). One could consider stopping after evaluating *n* irrelevant references or papers in a row. We recommend stopping when in the last *N*_*r*_ references, the number of new relevant additions *r*_*r*_ is less than some constant *C*, given that the number of snowballed papers *s* is at least *S*. For example, if our set of inclusions contains 100 papers, we may set the minimum number of papers to snowball to *S* = 10. Once 10 papers have been snowballed, we stop when the last *N*_*r*_ = 100 references contained less than *C* = 5 additions to our inclusions.

Although both backward snowballing and forward snowballing can be potentially relevant, we argue to only apply backward snowballing in SYMBALS. Given that grey literature and older papers will generally constitute the largest group of relevant papers not yet included, it is more apt to inspect references than citations. Forward snowballing is well suited to updating systematic reviews (Wohlin et al., 2020), but, as we show in Section 4.1.7, SYMBALS can also be used to update a systematic review.

### 3.5 Quality Assessment

From the core of SYMBALS, we now move back to traditional stages in systematic review methodologies. It is common to apply a quality assessment procedure to the research included after the completion of title and abstract screening (Kitchenham and Charters, 2007). It is certainly not a mandatory step in a systematic review (Brereton et al., 2007), nor is it a mandatory step.

Based on criteria for good practice (Kitchenham et al., 2002), the software engineering field outlines four main aspects of quality assessment: reporting, rigour, credibility, and relevance (Dybå and Dingsøyr). We believe these aspects to be broadly applicable. According to the specific needs of a systematic review, quality criteria can be formulated based on the four main aspects (Zhou et al., 2015).

No universally accepted quality assessment methodology exists (Zhang and Ali Babar, 2013). Automation of quality assessment is generally not even discussed. This highlights that there are possibilities to improve current quality assessment practice with machine learning techniques.

### 3.6 Data Extraction and Synthesis

Researchers should design data extraction and collection forms (Kitchenham and Charters, 2007) based on the research questions formulated during protocol development. These forms have the express purpose of helping to answer the research questions at hand but can also facilitate verifiability of the procedure. A well-designed data extraction form can even be made publicly available in conjunction with a publication (Morrison et al., 2018), to stimulate further research based on the results.

Data synthesis involves either qualitatively or quantitatively summarising the included primary studies (Kitchenham and Charters, 2007). Quantitative data synthesis, or meta-analysis, is especially useful if the extracted data is homogeneous across the included primary studies (Wohlin et al., 2012). Homogeneity can be promoted through a well-defined data extraction form. When performing a meta-analysis, researchers should be careful to evaluate and address the potential for bias in the primary studies (Wohlin et al., 2012), as this can threaten the validity of the results. It is recommended to include quality assessment results in the data synthesis phase, as it can offer additional insights into the results obtained by primary studies of varying quality.

### 3.7 Validation

The last step in our methodology is validation. Although validation is not explicitly included in all systematic review methodologies (Kitchenham and Charters, 2007; Wohlin et al., 2012), its importance is clearly recognised (Brereton et al., 2007; Moher et al., 2015). It is quite common for systematic reviews to assess the quality of primary studies based on whether limitations and threats to validity are adequately discussed (Zhou et al., 2015). We want to promote validation in systematic reviews themselves, which is why validation is a separate step in SYMBALS, rather than simply another reporting item.

There are four main validity categories: construct, internal, external, and conclusion (Zhou et al., 2016). We designed our methodology to counter threats to validity from all categories. Examples are unclear inclusion and exclusion criteria (Khan et al., 2019) and a subjective quality assessment (Zhou et al., 2016). Other commonly included elements during validation are an estimate of coverage of relevant research (Zhang et al., 2011) and an investigation of bias handling in data extraction and synthesis (Zhou et al., 2016).

The swiftness of our methodology allows us to introduce a new validation method in this paper: replication. An application of this novel validation method is presented in Section 4.1.7.

## 4 Case Studies

To assess the properties and the validity of our methodology, we performed two case studies. The first investigates the ability of SYMBALS to accommodate both broad coverage and a swift process. The second compares our methodology to the FAST^2^ (Yu and Menzies, 2019) methodology on four benchmark datasets. This allows us to evaluate both the effectiveness of our methodology in an absolute sense (case study 1) and relative to a state-of-the-art methodology (case study 2).

In both case studies, we used ASReview (van de Schoot et al., 2021) to perform title and abstract screening using active learning. Besides the fact that we found this tool to be easy to use, we applaud the commitment of the developers to open science and welcome their decision to make the codebase available open-source. Nonetheless, we want to stress that there are many other potent active learning tools available (Harrison et al., 2020).

As with most tools that support active learning for title and abstract screening, ASReview offers many options for the model to use (van de Schoot et al., 2021). We elected to use the default Naïve Bayes classifier, with TF-IDF feature extraction and certainty-based sampling. The authors state that these default settings produced consistently good results across many datasets (van de Schoot et al., 2021). Since Naïve Bayes is generally considered to be a relatively simple classifier, and the default feature extraction and sampling settings are available in most other active learning tools (van de Schoot et al., 2021), using these default settings facilitates reproducibility of our results.

### 4.1 Case Study 1: Cybersecurity Metric Research

The field of cybersecurity needs to deal with a constantly changing cyber threat landscape. Security practitioners and researchers feel the need to address this challenge by devising security solutions that are by their nature adaptable (Wang and Lu, 2018; Sengupta et al., 2020). This requires a corresponding adaptivity in cybersecurity research methods, which is why cybersecurity metric research is an appropriate domain to apply and examine our approach.

Although research into the measurement of cybersecurity risk has matured in past decades, it remains an area of fierce debate. Some researchers feel that quantified security is a weak hypothesis, in the sense that “it lacks clear tests of its descriptive correctness” (Verendel, 2009). Others feel it is challenging, yet feasible (Pfleeger and Cunningham, 2010). Yet others conjecture that security risk analysis does not provide value through the measurement itself, but through the knowledge analysts gain by thinking about security (Slayton, 2015). Nevertheless, the overwhelming consensus is that cybersecurity assessment is necessary (Jaquith, 2007).

Reviews are common in the cybersecurity metric field, but they are generally not systematic reviews. There are exceptions, although most are either outdated at this stage (Verendel, 2009; Rudolph and Schwarz, 2012), or only cover a specific area of cybersecurity, such as incident management (Cadena et al., 2020). In a particularly positive exception in the area of software security metrics (Morrison et al., 2018), the researchers did not only provide a clear explanation of their methodology but have also made their results publicly available and accessible. Still, there is a need for a broad systematic review in this area, and with this first demonstration and future research, we hope to build on initial positive steps.

In the interest of brevity, we will only cover those facets and findings of our application that are of general interest, leaving out specific details of this implementation.

#### 4.1.1 Develop and Evaluate Protocol

**FIGURE 4:**
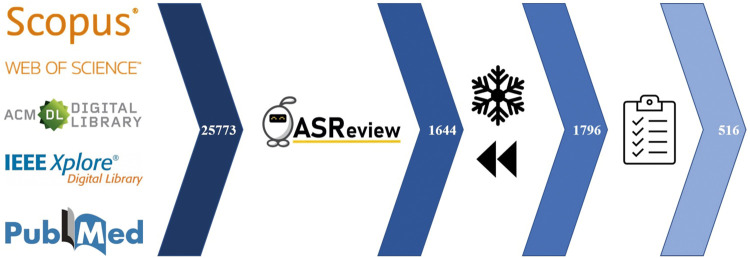
The SYMBALS implementation for the cybersecurity metric research case study. The database search, screening using active learning, backward snowballing, and quality assessment steps are shown, with the number of inclusions at each stage.

The first step in SYMBALS is to develop and evaluate a systematic review protocol. Our protocol was constructed by one researcher and evaluated by two others. Based on existing guidelines on relevant databases (Kitchenham and Charters, 2007), we selected the sources depicted in Figure 4. CiteSeerx and JSTOR were excluded due to the inability to retrieve large quantities of research from these sources. The search string selected for the Scopus database was:

AUTHKEY [(security* OR cyber*]

AND [(assess* OR evaluat* OR measur* OR metric* OR model* OR risk* OR scor*)]

AND LANGUAGE (english) AND DOCTYPE (ar OR bk OR ch OR cp OR cr OR re).

The asterisks denote wildcards. We only considered English language publications and restricted the search to articles (ar), books (bk), book chapters (ch), conference papers (cp), conference reviews (cr) and reviews (re).

#### 4.1.2 Database Search

The Scopus search string did not always translate well to other databases. This is a known issue (Singh and Singh, 2017) which we cannot fully circumvent, although a simpler search string helps to solve this problem. Other problems we encountered were that ACM Digital Library and IEEE Xplore limit the number of papers you can reasonably access to 2,000 and that IEEE Xplore only allows the use of six wildcards in a query. In the end, we chose to stick with our original query and sources, knowing that the active learning and snowballing phases would help in solving most of the potential issues. After cleaning and deduplication, 25,773 papers remained.

#### 4.1.3 Screening Using Active Learning

For the active learning phase, we used ASReview (van de Schoot et al., 2021). We elected to stop evaluating when 20 consecutive papers were marked irrelevant; a simple criterion similar to criteria used in earlier work (Ros et al., 2017). Figure 4 shows that 1,644 papers remained at the end of the active learning phase.

#### 4.1.4 Backward Snowballing

Next, we applied backward snowballing. We copied the evaluation order of the active learning phase. This is a simple and reproducible strategy, that we recommend others to follow when applying our methodology. We chose to stop when 10 consecutive papers contained no additions to our set of inclusions; a strict but simple criterion. If researchers are looking for an alternative strategy, we recommend considering a stopping criterion based on the inclusion rate over the last *N*_*r*_ references, where *N*_*r*_ is a predefined constant. An example of such a strategy is given in Section 3.4. The backward snowballing phase left 1,796 included papers.

#### 4.1.5 Quality Assessment

Given the large number of included papers at this stage, the logical choice was to apply a quality assessment step. We adapted the most relevant commonly used quality criteria (Zhou et al., 2015), to be suitable for use in combination with a Likert scale. Two researchers evaluated 40 papers each, with 20 of those papers being evaluated by both researchers. Table 2 shows the averaged results, where the scoring of the first researcher was used for the 20 duplicate papers.

**TABLE 2:** The quality criteria applied to 60 papers during the quality assessment phase. The most commonly used criteria (Zhou et al., 2015) were assessed for relevance. The most relevant criteria were reformulated to be suitable for use in combination with a Likert scale. Statements could be responded to with strongly disagree (SD), disagree (D), neutral (N), agree (A), or strongly agree (SA).

Aspect	Criterion	SD	D	N	A	SA
Reporting	There is a clear statement of the research aims	0	4	7	28	21
Reporting	There is an adequate description of the research context	0	6	11	17	26
Reporting	The paper is based on research	0	3	3	16	38
Rigour	Metrics used in the study are clearly defined	0	10	19	16	15
Rigour	Metrics are adequately measured and validated	1	24	22	8	5
Rigour	The data analysis is sufficiently rigorous	0	21	17	14	8
Credibility	Findings are clearly stated and related to research aims	0	8	19	25	8
Credibility	Limitations and threats to validity are adequately discussed	30	18	8	2	2
Relevance	The study is of value to research and/or practice	0	9	12	28	11

The response to each quality criterion was scored with 0, 0.25, 0.5, 0.75 or 1, corresponding to the five possible evaluations. With the sheer size of the set of inclusions, it was not possible to assess the quality of all papers. One possible solution to this problem is the following. We split the 60 evaluated papers into a training set (48 papers) and a test set (12 papers). Each paper was labelled as having sufficient quality if it obtained a score of at least 6 out of 9. In the 20 papers that were evaluated by both researchers, there were five edge cases where a disagreement occurred. On average, the quality scores differed by roughly 0.7 points. The researchers were almost equally strict in the evaluation of the papers, with the total sum of all quality scores differing by just 0.25.

We extended our quality scores with three explanatory features: years since publication, citation count, and the number of pages. A binary decision tree was trained on the explanatory features for the 48 training papers and evaluated on the 12 test papers. The model predicted 11 of the 12 papers correctly, incorrectly predicting one edge case with a quality score of 6 as having insufficient quality.

This short demonstration shows that training decision trees on assessed papers is a viable alternative to other strategies to filter a large set of inclusions. Commonly used alternatives are to only consider articles or to limit the time frame of the search. A decision tree trained on actual researcher quality assessments is an interesting substitute for traditional approaches, although we wish to stress that it is fully up to researchers using SYMBALS to choose which approach they apply. Additionally, quality assessment is an optional phase in SYMBALS, meaning researchers could even choose to not apply this step.

#### 4.1.6 Data Extraction and Synthesis

**FIGURE 5:**
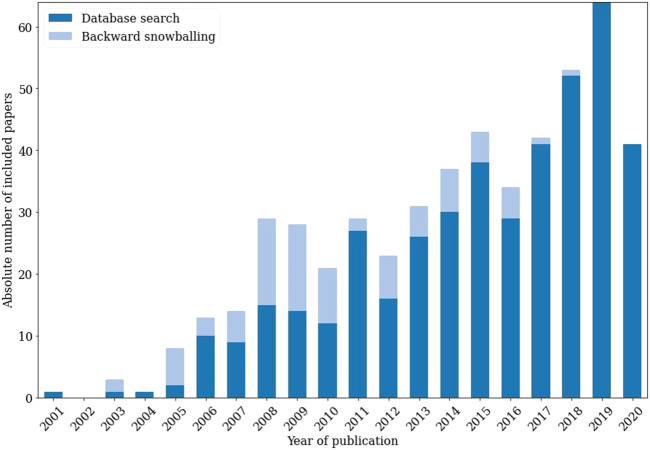
The absolute number of cybersecurity metric papers per year in the final inclusion set. We distinguish papers resulting from database search (dark) from those resulting from backward snowballing (light). For papers from 2010 and earlier, 45% originated from backward snowballing.

After applying the resulting criteria of the decision tree to our inclusions, the 516 inclusions indicated in Figure 4 remained. The set of excluded papers comprised both research that did not pass the decision tree assessment and research that had insufficient data for assessment. Figure 5 illustrates the importance of the backward snowballing phase. Of our inclusions, 17% originated from backward snowballing. Considering only papers from before 2011, this figure jumps to 45%, highlighting the potential weakness of using only a database search step. Figure 5 therefore demonstrates the ability of SYMBALS to ensure broad coverage over time.

After an initial analysis of our inclusions, we formulated our data extraction form and used this as a guide to extract the necessary data. We then used quantitative data synthesis to produce more detailed and insightful results, aided by the homogeneity of our extracted data. Given that this is a demonstration of our methodology, rather than a complete systematic review study, we leave further analysis and presentation of our detailed results for future work.

#### 4.1.7 Validation

To validate our case study, as well as the methodology itself, we performed a replication experiment. We extended the existing review with research from the months following the initial database search, using the same initiation process and stopping criteria as defined in Sections 4.1.3 and 4.1.4. The replication was performed by both the main researcher and a researcher who was not involved in the initial review. This allowed us to answer the question of whether SYMBALS contributes to an accessible and swift process.

The database search procedure uncovered 2,708 papers, of which 222 were evaluated in the active learning phase. In the backward snowballing phase the main researcher evaluated 300 references. A common estimate for the time taken to screen a title-abstract record is a minute (Shemilt et al., 2016). This aligns with our time spent on the screening phase, which was 4 h (222 min is 3.7 h). The average time to scan one reference during backward snowballing can be expected to be lower than a minute, since a certain portion of the references will either have been evaluated already or will be obviously irrelevant (e.g., website links). Our backward snowballing phase took 3.5 h, which corresponds to 0.7 min per reference. Altogether the process took 7.5 h, whereas screening the titles and abstracts of 2,708 papers would have taken over 45 h. Hence, we were able to speed up the title and abstract screening phase by a factor of 6.

To address the question of accessibility, we asked a researcher that had not been involved in the review to also perform the replication experiment. After 2 h of explanation, the researcher was able to complete the active learning and snowballing phases, albeit roughly 3 times as slow as the main researcher. Note that this is still twice as fast as the traditional process. Automatic exclusion during active learning contributes to this speed. However, given the relatively short time that was required to explain the methodology, we argue that the structure SYMBALS offers is another reason that it accommodates a swift process.

An additional element that is worth addressing is trust in the active learning process (O’Connor et al., 2019). One question that hovers over machine learning techniques is whether their random elements negatively impact reproducibility. To test this statement for the ASReview tool, we investigated how the first 100 papers of the active learning phase would change under different levels of disagreement with the main researcher. Our ASReview process starts after presenting five prior relevant papers to the tool and evaluating five random papers. In our first experiment, we copied all earlier decisions by the main researcher. This already resulted in small changes to the order in which papers were recommended. This poses a problem when using our stopping criterion, as changes in the order can alter the moment at which a researcher has reached *n* consecutive irrelevant papers. This is one of the reasons we recommend using more sophisticated stopping criteria.

The changes in order persisted when for 20% of the papers the initial evaluation of the main researcher was reversed. In both cases, the changes in order were minimal for the first 20 papers. This is important, as these papers will be the first papers considered in the backward snowballing phase. The replication of the second researcher had an even higher level of disagreement in the first 100 papers of 37%, which was a natural consequence of differing experience in the cybersecurity metrics field. Interestingly, even with this level of disagreement, the first 17 papers did not contain a paper outside of the first 25 papers of the main researcher. We believe this shows that the process is robust to inter-rater disagreement, given the correct stopping criterion.

### 4.2 Case Study 2: Benchmarking

Besides evaluating the performance of our methodology in an absolute sense, we additionally evaluated its performance compared to an existing state-of-the-art methodology. We benchmarked the SYMBALS methodology using datasets (Yu et al., 2020) developed for the evaluation of the FASTREAD (Yu et al., 2018b) and FAST^2^ (Yu and Menzies, 2019) systematic review methodologies. The datasets of both inclusions and exclusions were constructed based on three systematic reviews (Wahono, 2007; Hall et al., 2012; Radjenović et al., 2013) and one tertiary study (Kitchenham et al., 2010).

In our benchmarking, we compare to the results obtained by the FAST^2^ methodology, since it is an improvement over the FASTREAD methodology (Yu and Menzies, 2019). For the three systematic reviews (Wahono, 2007; Hall et al., 2012; Radjenović et al., 2013), the authors reconstructed the datasets based on information from the original papers. For the tertiary study (Kitchenham et al., 2010), the dataset was provided by the original authors of the review. The reason that we chose to compare to FAST^2^ is not only because it is a state-of-the-art methodology, but also because the FAST^2^ datasets were so easily accessible and in a compatible format for SYMBALS. This was not the case for the other methodologies covered in Table 1, such as Mourão et al. (2017, 2020).

SYMBALS and FAST^2^ cannot be fairly compared without first adjusting the datasets. After a database search, the FAST^2^ method uses active learning as the sole approach for title and abstract screening. In the FASTREAD and FAST^2^ papers, the authors make the necessary assumption that the datasets encompass all relevant papers since these methodologies have no way of discovering relevant research outside of the original dataset. However, in research that incorporates snowballing in systematic reviews, it has been shown that between 15 and 30% of all relevant papers are not included in the original dataset (Mourão et al., 2017, 2020). This aligns with our results in the first case study, where 17% of the inclusions originated from backward snowballing.

To enable a fair comparison of SYMBALS and FAST^2^, we randomly removed 15% of both the relevant and irrelevant papers in the datasets before initiating our active learning phase. The removed papers were then considered again in the backward snowballing phase of SYMBALS. This adjustment allows our benchmarking study to accurately reflect the actual situation faced by researchers performing systematic reviews. The consequence of this adaptation is that the recall achieved by the FAST^2^ methodology is multiplied by a factor of 0.85.

Both the FASTREAD and FAST^2^ papers address the definition of an initiation process and a stopping criterion. Regarding initiation, two approaches are posited: “patient” and “hasty.” The patient approach generates random papers and initiates active learning once five inclusions are found. The hasty approach initiates active learning after just one inclusion is found. To leave room for the backward snowballing phase, we used the hasty method for initiation.

Many of the stopping criteria considered in FAST^2^ cannot be applied in our setting, since they rely on properties of the specific active learning tool used for the methodology. To ensure a transparent approach, we opted to stop after 50 consecutive exclusions. This stopping criterion, sourced from earlier work (Ros et al., 2017), was found to yield the fastest active learning phase on average in the FAST^2^ paper. This is useful in our setting, as it again leaves time for the backward snowballing phase.

**FIGURE 6:**
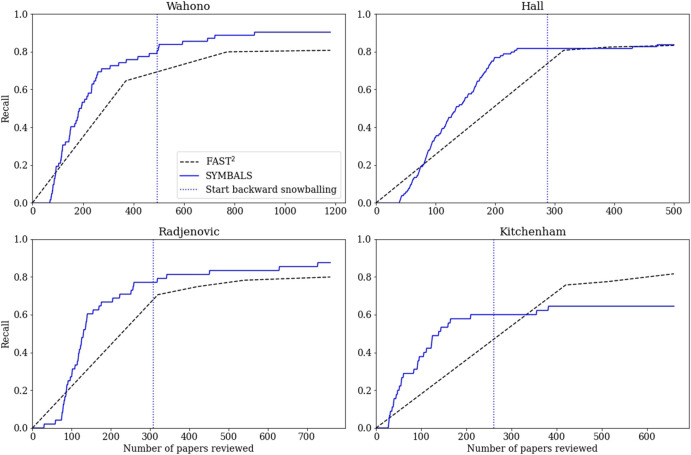
The recall achieved by the FAST^2^(Yu and Menzies, 2019) and SYMBALS methodologies, for the four review datasets studied in our benchmarking case study. For the FAST^2^ method we provide linear interpolations of the median results. A vertical dotted line indicates the start of the backward snowballing phase for SYMBALS.

We conducted the active learning phase of our benchmarking experiments using the ASReview tool (van de Schoot et al., 2021) that we also used in our first case study. The results are shown in Figure 6. As mentioned before, the recall achieved by the FAST^2^ methodology was multiplied by a factor of 0.85, to align with the removal of 15% of the papers.

The FAST^2^ results are linear interpolations of the median results provided by the authors in their paper. For the later data points, this linear extrapolation represents the actual data with reasonable accuracy. However, for the earlier data points, the linear extrapolation overestimates the recall achieved by FAST^2^. FAST^2^, like SYMBALS, takes time to find the first few relevant papers, due to the nature of the applied initiation process. This observation is confirmed when examining the graphs presented in the FAST^2^ paper. Although the overestimation of recall in the early phase is not ideal for our comparison, we are mainly interested in how the methods compare beyond initiation. We employ the same initiation process as FAST^2^, meaning differences in performance during the initiation phase are purely due to random deviations.

For the three traditional systematic review papers (Wahono, 2007; Hall et al., 2012; Radjenović et al., 2013), our methodology achieved a higher recall than FAST^2^. At the maximum number of reviewed papers, SYMBALS achieved a 9.6% higher recall for the Wahono dataset (90.3% compared to 80.7%), a 0.4% higher recall for the Hall dataset (83.7% compared to 83.3%), and a 7.6% higher recall for the Radjenovic dataset (87.5% compared to 79.9%). In all three of these cases, the active learning phase of SYMBALS performed well, achieving a recall higher than the recall of FAST^2^ after evaluating the same number of papers. Nevertheless, in each case, the recall achieved after the active learning phase was lower than the eventual recall of FAST^2^.

The backward snowballing phase of our methodology raised the recall achieved in the active learning phase by 9.7% for the Wahono dataset, by 1.9% for the Hall dataset, and by 10.4% for the Radjenovic dataset. At first, these contributions may seem to be minor. However, as recall increases, further improving recall becomes increasingly difficult. In light of this observation, the backward snowballing additions are the key element in ensuring that SYMBALS outperforms FAST^2^ for the Wahono, Hall, and Radjenovic datasets. Considering the finding from our first case study that reviewing references during backward snowballing is faster than screening titles and abstracts during active learning, SYMBALS achieves a higher recall in less time than FAST^2^.

For the tertiary study (Kitchenham et al., 2010), the performance of SYMBALS (64% recall) was relatively poor compared to FAST^2^ (82% recall). Both the active learning phase and the backward snowballing phase underperformed compared to the other studies. Regarding the active learning phase, one explanation could be that the content of the titles and abstracts were not identifiably different for relevant and irrelevant papers. This is certainly a plausible scenario given that the tertiary study screens systematic reviews, which are likely to differ more in their content than regular papers aimed at a specific topic. This does not explain, however, how FAST^2^ was able to achieve a high recall. The difference between the performance of ASReview and the active learning of FAST^2^ is a consequence of algorithmic differences, but these algorithmic differences were not investigated further.

It is not surprising that backward snowballing is less useful for tertiary studies, as the systematic reviews that they investigate are less likely to reference each other. Furthermore, systematic reviews often have many references. The 400 references we evaluated for the tertiary study, came from just five papers. With fewer papers to investigate, the scope of the backward snowballing phase is narrowed. A final factor that may have influenced results, is that the authors of the tertiary study explicitly focus on the period between the 1st of January 2004 and the June 30, 2008. A short timespan restricts the effectiveness of backward snowballing.

We believe this benchmarking study highlights the areas where our approach can improve upon existing methodologies. When researchers are looking to systematically review research over a long period, SYMBALS can trump state-of-the-art methodologies on their home turf. When researchers are interested in additionally including grey literature or expect that not all relevant papers are included in their initial dataset, our methodology offers further advantages through the inclusion of a backward snowballing step. When researchers are performing a tertiary study, fully automated methods such as FAST^2^ may be more appropriate than SYMBALS. Future research employing and evaluating our methodology will help to further clarify its strengths and weaknesses.

## 5 Discussion and Limitations

We posed the following research question at the outset of this paper: How can active learning and snowballing be combined to create an accessible and swift systematic review methodology? The review of existing research in systematic review methodologies and active learning in Section 2, combined with the additional analysis in Sections 3.3 and 3.4, helped us to formulate a methodology inspired and motivated by existing work. Figure 2 outlines the resulting proposal. We found that active learning is best suited to the screening of titles and abstracts and that backward snowballing provides an ideal supplement. The combination facilitates coverage of relevant (grey) literature while maintaining a reproducible procedure.

In the case study of Section 4.1, 17% of the relevant research would not have been found without backward snowballing. This figure jumps to 45% when only considering research from before 2011. We further investigated the properties of our methodology in Section 4.1.7. The fact that a researcher who was new to the case study review was able to execute our methodology after just 2 h of explanation, shows that it is easily understandable and accessible. Moreover, SYMBALS was shown to accelerate title and abstract screening by a factor of 6, proving that it accommodates a swift procedure through its active learning component.

Section 4.2 compared the performance of our approach to the state-of-the-art systematic review methodology FAST^2^ (Yu and Menzies, 2019). We found that SYMBALS achieves a 6% higher recall than FAST^2^ on average when applying the methodologies to systematic reviews. FAST^2^ was found to outperform SYMBALS for a tertiary study benchmark, pointing to a possible case where SYMBALS may not be the most suitable methodology.

Our methodology has its limitations. The lack of trust in systematic review automation technologies (O’Connor et al., 2019) is not fully solved by SYMBALS. Active learning methods and tools have matured, but there will still be researchers who feel uncomfortable when applying them in reviews. This limits the use of our approach to only those researchers who trust the automation technologies employed. Likewise, practical limitations exist. Depending on the exact implementation, researchers will have to have some computer programming skills. ASReview, for example, requires the installation and use of the ASReview Python package. The heterogeneity of online databases is another limitation our methodology cannot fully address, although the fact that SYMBALS allows researchers to avoid complex search queries partially counters this issue.

Lastly, we should address potential threats to validity. A handful of researchers evaluated SYMBALS throughout this process. Although their varying experience levels and areas of expertise allowed us to address questions of accessibility and reproducibility, we admit that in the future more evaluation is desirable. Another potential pitfall is the quality of abstracts in fields outside the fields considered in our case studies. There are areas of research where it is known that abstract quality can be poor (Brereton et al., 2007). This can potentially harm the effectiveness of active learning in abstract screening. Altogether, we believe that the benefits of SYMBALS far outweigh its limitations, which is why we strongly believe it can have a lasting impact on the systematic review landscape.

## 6 Conclusion and Future Research

This paper introduced SYMBALS: a SYstematic review Methodology Blending Active Learning and Snowballing. Our methodology blends the proven techniques of active learning and backward snowballing to create an effective systematic review methodology. A first case study demonstrated the ability of SYMBALS to expedite the systematic review process, while at the same time making systematic reviews accessible. We showed that our approach allows researchers to accelerate title and abstract screening by a factor of 6. The need for backward snowballing was established through its contribution of 45% to all inclusions from before 2011. In our benchmarking study we demonstrated the ability of SYMBALS to outperform state-of-the-art systematic review methodologies, both in speed and accuracy.

In future research, we hope to further evaluate and validate our methodology, including the completion of the full cybersecurity metric review case study. Another interesting avenue for future research is to investigate which choices in the selection of active learning tools, classification models, and stopping criteria are optimal in which scenarios. Optimising SYMBALS in these areas can certainly benefit researchers performing systematic reviews, although they should take care to not reduce the reproducibility of their results.

Finally, we believe that there are promising possibilities for further systematic review automation. Machine learning techniques and opportunities exist for all areas of the systematic review procedure. As these techniques mature, we will see an increase in their use. Research into how to incorporate these techniques in systematic review methodologies in a way that harbours trust, robustness, and reproducibility, is of paramount importance. We hope that SYMBALS is the next step in the right direction.

## Data Availability

The raw data supporting the conclusions of this article will be made available by the authors, without undue reservation.
